# The critical role of coastal protected areas in buffering impacts of extreme climatic conditions on bird diversity and their ecosystem services' provisioning in the Eastern Cape Province, South Africa

**DOI:** 10.1002/ece3.10452

**Published:** 2023-10-20

**Authors:** Thabiso Michael Mokotjomela, Loyd Rodney Vukeya, Lwandiso Pamla, Zimbini Scott

**Affiliations:** ^1^ South Africa National Biodiversity Institute Free State National Botanical Garden Bloemfontein South Africa; ^2^ School of Life Sciences University of KwaZulu‐Natal Pietermaritzburg South Africa; ^3^ Free State National Botanical Garden Bloemfontein South Africa; ^4^ Scientific Services Unit Eastern Cape Parks and Tourism Agency East London South Africa

**Keywords:** allometry, birds, conservation, ecological services

## Abstract

In this study, we documented the diversity of bird species in the Eastern Cape coastal nature reserves (i.e., Hluleka, Dwesa, Silaka and Mkhambati nature reserves), and determined the potential role of each bird species in habitat maintenance using two functional traits (i.e., body mass and feeding mode) as the function's proxy. We applied the timed species count approach during bird observations, coupled with drive‐by surveys to maximise spatial coverage of each nature reserve over four years. To evaluate functional diversity, bird species were classified based on functional traits such as the adult body, and their potential ecological role derived from their feeding mode and habitat associations. Over 864 h, we accumulated 818 bird records containing 178 different bird species that were classified into 58 families with 32 species occurring in all nature reserves. Shannon–Wiener Diversity Indices showed very high overall species diversity across the nature reserves (*H* > 3.5) with no differences detected across sites. Although no significant correlations between vegetation changes measured through Normalised Difference vegetation Index (NDVI) in each nature reserve and the number of bird records, forest bird species were dominant (42.1%; *N* = 178) throughout years of observation and diversity remained high (*H* > 3.5). Bird species abundance only increased significantly across all nature reserves during 2018–2019. All four nature reserves had a similar distribution of bird functional traits with both high functional richness (FRic = 1), and divergence (FDiv = 0.8) and moderate evenness (FEve = 0.4). Multiple Correspondence Analysis (MCA) demonstrated a positive correlation between bird sizes and functions with large birds mainly associated with predators and carrion. Small birds and medium birds had a similar composition of species in terms of functionality being seed dispersers across the nature reserves. A significant effect that insectivores and carrions displayed in MCA plots, suggest the availability of indirect pollination services. Despite extreme drought conditions across the country in 2019, NDVI levels remained largely consistent over time in these four reserves; and thus, they offer important refuge for birds during extreme climatic conditions such as drought.

## Graphical abstract

Over 864 h of field sampling in 2017–2020, we accumulated 818 bird records containing 178 different bird species classified into 58 families. Shannon–Wiener Diversity Indices showed very high overall species diversity, and across the nature reserves (*H* > 3.5) with however, all nature reserves being non‐significantly different. However, the year 2018/2019, showed significantly higher abundance of birds across all nature reserves. Among different feeding modes, potential seed dispersers had a significantly greater number of records than other groups while the pollinators were not significantly different in number compared to seed predators.

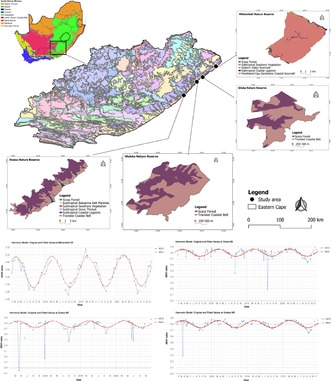

## 1 INTRODUCTION

Globally, protected biodiversity conservation areas, which include both natural and cultural heritage conservation (SCBD, 2004), are regarded as one of the most effective tools for protecting natural resources (Muhumuza & Balkwill, 2013; Possingham et al., 2006; Schulze et al., 2017; Watson et al., 2014, 2018). Of great importance is the network of protected areas in South Africa, reported to sustain a relatively high abundance of bird species (Duckworth & Altwegg, 2018). Birds provide important ecological services for maintaining ecosystems and supporting biodiversity, especially during interactions with plants (Şekercioğlu et al., 2016; Whelan et al., 2008). However, those ecological services are threatened by human disturbance and climate change (Gomes et al., 2008; Grobler & Campbell, 2022). For example, frugivorous birds avoiding foraging in highly disturbed habitats, which could jeopardise the ecological services they provide (Grobler & Campbell, 2022).

The interest in associations between various habitat types and bird species' diversity has gained importance as a critical issue in biodiversity conservation (Mao et al., 2023; Tu et al., 2020) possibly because species diversity or richness can predict the functional diversity in a habitat (Martin et al., 2019; Seymour et al., 2015; Tilman, 2001). Functional diversity measures either the diversity of functionally different species with their different functional traits in the properties of a community (Martin et al., 2019; Petchey et al., 2004; Silva et al., 2020; Suárez‐Castro et al., 2022; Tilman, 2001), and it is important for understanding the dynamics and architecture of many ecosystems (Gagic et al., 2015; Mao et al., 2023; Tilman, 2001). It is suggested that functional diversity connects organisms and ecosystems to better explain and predict ecological processes maintaining the habitat (Li et al., 2022; Petchey et al., 2004). Often, high functional diversity is linked to the high occupation of niche space, which buffers against the addition of new functional groups including alien species in a habitat (Rejmanek, 1996), while low functional diversity in a habitat is generally associated with poor resistance to invasion by new species (Levine et al., 2004). Consequently, the protected biodiversity areas not only pursue maintenance of high taxonomic richness but also functional diversity as a way of increasing habitat resilience and stability (Cottee‐Jones et al., 2015; Lotz, 2021). For example, studies demonstrate that often, bird species' richness is positively influenced by the availability of major habitat types and their ecological integrity within each ecosystem (Leveau, 2019; Rafe et al., 1985). Forest habitats are consistently shown to support more bird species than other habitat types because of their structural and resources' diversity which are integral for birdlife during different seasons of the year (Cooper et al., 2017; Oatley, 1989). The high ecological diversity in a local habitat is a positive driver of habitat productivity and, thus, the persistence of habitat functionality (Şekercioğlu et al., 2016; Tilman et al., 1997; Whelan et al., 2008). On the contrary, it is well known that habitat loss through anthropogenic disturbance reduces habitat quality and results in a dramatic decline in bird species' diversity (Gomes et al., 2008; Leaver et al., 2019; Wilms & Kappelle, 2006). In Ethiopia, Yineger and Hughes (2014) reported a significant decline in forest specialists, insectivores, frugivores and open‐nester bird species because of forest loss and fragmentation driven by anthropogenic habitat degradation. Conversely, other studies have found that biophysical distance associated with urbanisation increased bird species' richness because residential gardens become foraging hotspots rich in alien plant food resources for birds (Blair, 1999; Mokotjomela et al., 2013b, 2022; Shochat et al., 2010; Tratalos et al., 2007). Therefore, documenting bird species and functional diversity as an indicator of environmental change is important (Gregory et al., 2003), especially in protected areas, because this can inform conservation planning and intervention efforts on changing ecosystem services provided by birds under global climate change.

The distribution of pollinators in the landscape can have important implications for plant fecundity (Johnson et al., 2009). Pollination is a critical fertilisation process that determines the viability of the seeds produced, which then influences plant recruitment (Stiles, 1981). Many birds provide pollination services to 5% of the native flora in South Africa (Geerts, 2016; Johnson, 2010; Johnson et al., 2009; Whitehead, 2018). Van der Niet et al. (2015) showed that specialised bird pollinators (e.g., sunbirds) support fruit production of the critically endangered species *Satyrium rhodanthum* (Orchidaceae) in the mistbelt grassland. Fang et al. (2012) similarly reported that avian pollinators (*Pycnonotus sinensis* and *Zosterops japonicus*) play a critical ecological role for winter‐flowering plant species when the co‐pollinators' insects are scarce because of cold winter temperatures in central China. The ornithophilous plants produce attractive tubular flowers that promote visitation frequency by potential bird pollinators (Van der Niet et al., 2014, 2015). These flowers reportedly offer nectar, especially to the often‐narrow guild of long‐billed specialist nectar‐feeding birds (Maruyama et al., 2014; Rebelo, 1987; Stiles, 1981). Indeed, trait‐matching to ecological roles played by certain bird species to their mutualistic partner plant species have been reported and is well‐known (Herrera, 1984; Horak & Janecek, 2021; Jordano, 1987). For example, pollinator sunbirds have evolved long bills that can maximise the extraction of nectar from hollow and nectar‐rich flowering species (Geerts & Pauw, 2009; Horak & Janecek, 2021; Janeček et al., 2011).

Frugivorous and granivorous birds are important seed dispersers of many plant species bearing fruits and seeds (Howe & Smallwood, 1982; Jordano, 2000; Mokotjomela et al., 2013a, 2013b, 2015; Schupp et al., 2010; Vukeya et al., 2022; Wang & Smith, 2002). Although granivorous birds are generally considered as seed predators, Mokotjomela et al. (2015) demonstrated their efficiency in the seed dispersal of Acacia species in South Africa. Previous studies have shown the advantages of bird‐mediated seed dispersal to support the persistence of local plant species' populations (Egerer et al., 2018; Howe & Smallwood, 1982). With bird‐mediated dispersal processes, seeds escape density‐dependent competition to safe microsites (Vukeya et al., 2021, 2023) and seed germination is also enhanced as a result of the bird's gut treatment (Mokotjomela et al., 2021; Nathan, 2007; Schurr et al., 2009; Tsoar et al., 2011; Vukeya et al., 2021). Frugivorous birds typically have short bills and an alimentary canal structure that minimises the damage of seeds during feeding on fruits (Jordano, 1987, 2000). Nevertheless, Egerer et al. (2018) found that the loss of frugivorous birds because of an invasive snake *Boiga irregularis* in the Mariana Islands led to the decline of a socially valued plant, *Capsicum frutescens*.

Allometry explicitly relates variation in life histories to the body size of an organism; and for birds, the most useful measure of body size is adult body mass (Dunning, 2018). Allometric relationships suggest that animal activity such as foraging associated with pollination, and seed dispersal services, is positively correlated to body mass (Calder, 1996; Mokotjomela et al., 2015; Schurr et al., 2009; Tsoar et al., 2011). Schurr et al. (2009) reported a high correlation between bird body mass, flight speed, seed load and seed passage time through the gut. Large birds may retain more seeds for longer periods in their guts and flew longer distances than small birds (Jordano, 2007; Mokotjomela, 2012; Mokotjomela et al., 2013b; Tsoar et al., 2011), with exceptions when birds consumed laxative alien fruits (Mokotjomela et al., 2015). However, small birds tend to cover large distances when avoiding dangerous events such as fires (Gomes et al., 2008; Mokotjomela et al., 2013a, 2013b; Wilms & Kappelle, 2006) and when tracking fruit resources (Berthold, 1999; Saracco et al., 2004; Telleria et al., 2008).

Alternatively, some bird species (granivores) are seed predators as they grind up entire seeds in muscular gizzards to optimise their nutritional value (Heleno et al., 2010; Hulme & Benkman, 2002; Kleyheeg et al., 2018; Ruxton & Schaefer, 2012). However, it has also been shown that some bird seed predators can increase plant fitness, especially in large‐seeded plants (Ruxton & Schaefer, 2012; Tella et al., 2016, 2019). Mokotjomela et al. (2015) showed that some seeds survive gut passage using captive doves (granivores: Laughing Dove *Streptopelia senegalensis* and Red‐eyed Dove *Streptopelia semitorquata*) during feeding trails: 18.8 ± 3.3% seeds retrieved were recognisable and had better germination after ingestion by doves in South Africa. Similarly, Tella et al. (2019) investigated seeds dispersed (*Araucaria araucana* and *Araucaria angustifolia*) by parrots in Australia and South America and found that the dispersed seeds can germinate faster after partial predation by parrots. By reducing the number of deposited seeds, seed predation may provide selective pressure for plant population maintenance in the local habitat (Mokotjomela et al., 2015), particularly in small‐seeded plants during drought conditions (Mendoza & Dirzo, 2007).

The Eastern Cape Province in South Africa is rich in biodiversity, containing seven centres of plant endemism (Skowno et al., 2019; Van Wyk, 1996; Van Wyk & Smith, 2001). The Province has ~665 bird species, including 35 globally threatened species (Lepage, 2020). Since birds are easy to identify in the field and are sensitive to disturbance (Gregory & van Strien, 2010), they are considered important indicators of biodiversity and environmental changes (Gomes et al., 2008; Gregory et al., 2003). The Eastern Cape Parks and Tourism Agency is managing the majority of the provincial protected biodiversity areas' network (~579,854 ha) (Skowno et al., 2012). With the growing climate change threats to biodiversity and poverty in South Africa (Mbendana et al., 2019), the biodiversity economy is a promising sector for future socio‐economy development because South Africa has rich biotas that can be explored with minimal capital investment (NBES, 2015). While nature‐based tourism is the main activity in the parks and nature reserves in the Eastern Cape Province (Mokotjomela & Nombewu, 2019), avi‐tourism is believed to have the potential to bolster the generation of revenue in nature‐based tourism (Mossaz et al., 2015). However, there has been limited attention to the documentation of local bird species for this purpose, possibly because of a high focus on game‐viewing in the province, particularly on rare large mammals (Mossaz et al., 2015). This knowledge gap undermines the importance of the role that birds play in habitat maintenance and thus may retard the inclusion of birds in whole‐habitat conservation efforts (Mossaz et al., 2015; New, 2017), with a specific focus on bird species suffering from home range decline (Mulvaney, 2021). Indeed, Cooper (2015) reported that half of South Africa's forest‐dependent bird species have declining ranges, with the loss of these species most prominent in the Eastern Cape Province. The whole‐habitat conservation concept (also called Ecosystem Approach) emphasises knowledge of biological species in the system and understanding of their interactions as well as their role in habitat maintenance (SCBD, 2004).

In view of this, the study aimed to document bird species' diversity in the Wild Coast nature reserves of South Africa (i.e., Mkhambati, Hluleka, Silaka and Dwesa‐Cwebe), and to determine the possible ecological role of each bird species in habitat maintenance using two functional traits: bird feeding mode and body mass classifications as a species function's proxy. Previous studies focusing conservation of birds (e.g., forest birds) confirmed that many bird species in the Eastern Cape Province, South Africa are threatened by habitat degradation (Cooper, 2015; Leaver, 2020; Mulvaney, 2021) and thus it is critical to assess the possible ecological interactions. It is possible that their associated ecosystem goods and services are also changing and their implications may be noted on vegetation in the long term. Indeed, it has been shown that knowledge of bird species' presence/absence in a habitat is crucial for conservation planning (BirdLife International, 2022; Manu et al., 2010).

## 2 MATERIALS AND METHODS

### 2.1 Study areas

**FIGURE 1:**
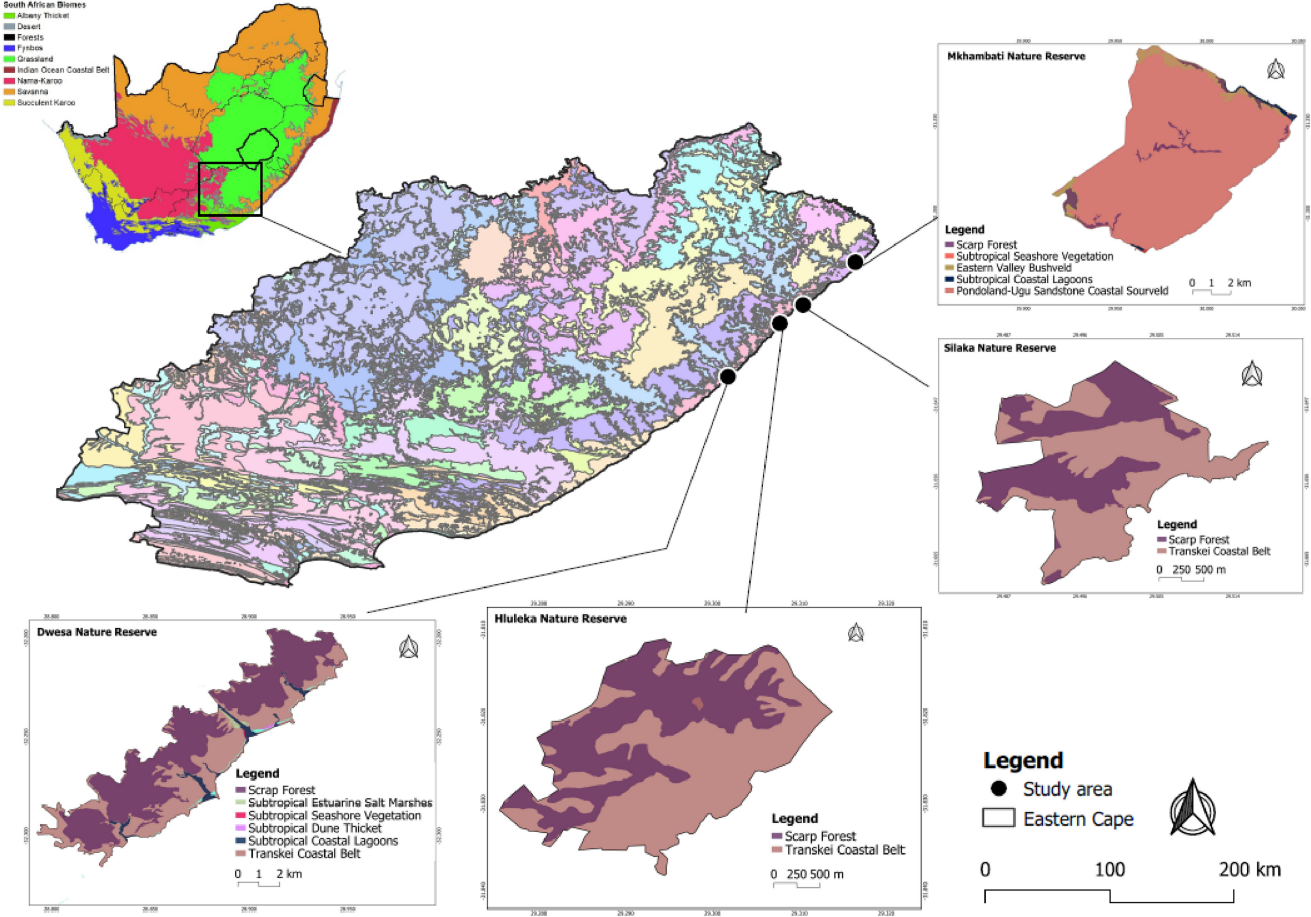
Map of the four nature reserves located on the Wild Coast in the Eastern Cape Province of South Africa. Different vegetation types for each nature reserve have been depicted with different colours.

We conducted our study in four protected areas—Mkhambati Nature Reserve (31°17′11.8″ S, 29°58′14.8″ E), Hluleka Nature Reserve (31°49′19.4″ S, 29°17′53.5″ E), Silaka Nature Reserve (31°39′16.0″ S, 29°30′05.3″ E), and Dwesa‐Cwebe Nature Reserve (32°15′26.3″ S, 28°52′06.0″ E)—located in two centres of plant endemism in the wild coast of Eastern Cape Province, South Africa: the Pondoland Centre and Maputaland–Pondoland Regions (Van Wyk, 1996; Van Wyk & Smith, 2001; Figure 1). The Pondoland Centre and Maputaland–Pondoland Regions possess a great potential to promote ecotourism because of the scenic beauty and rich biodiversity.

According to Mucina and Rutherford (2006), the eastern wild coast area comprises open grasslands and six different forest types which include Pondoland Coastal Forest, Dune Forest and Swamp Forest (Figure 1 and Table 1). The grassland vegetation is dominant in the protected areas and provides essential breeding sites for birds (Maphisa et al., 2016). Similarly, the Coastal Forest provides a variety of food resources and nesting sites for different organisms (Olivier & van Aarde, 2017). In South Africa, both grassland and forest vegetation types face a range of environmental threats such as uncontrollable fires, overgrazing, overharvesting, invasive alien species and climate change (Lechmere‐Oertel, 2014; Skowno et al., 2019; Stavi, 2019).

**TABLE 1:** Nature reserves of the study area, with the total reserve size coverage, biomes and vegetation types (adapted from Mucina & Rutherford, 2006).

Nature reserves	Reserve size (ha)	South African biome	Vegetation types
Mkhambati	7736.19	Savanna, Forestry, Azonal Vegetation and Indian Ocean Coastal Belt	Eastern Valley Bushveld (SVs6)—%, Pondoland‐Ugu Sandstone Coastal Sourveld (CB4)—91%, Scarp Forest (FOz5) 4%, Subtropical Coastal Lagoons—0.8% and Subtropical Seashore Vegetation (AZd4)—0.2%
Hluleka	576.53	Indian Ocean Coastal Belt and Forestry	Scarp Forest (FOz5)—44% and Transkei Coastal Belt (CB5)—56%
Silaka	400.33	Indian Ocean Coastal Belt and Forestry	Scarp Forest (FOz5)—41% and Transkei Coastal Belt (CB5)—59%
Dwesa	5528.83	Indian Ocean Coastal Belt, Azonal Vegetation, and Forestry	Scarp Forest (FOz5)—61.7%, Subtropical Coastal Lagoons—0.2%, Subtropical Dune Thicket (AZs3)—0.1%, Subtropical Estuarine Salt Marshes (AZe3)—3%, Subtropical Seashore Vegetation, (AZd4)—1%, and Transkei Coastal Belt (CB5)—34%

Mkhambati Nature Reserve: Approximately 91% of the reserve is dominated by Pondoland–Natal Sandstone Coastal Sourveld grassland (Dayaram et al., 2019; Skowno et al., 2019). Other vegetation types found in the reserve are the Eastern Valley Bushveld, Scarp Forest, Subtropical Coastal Lagoons and Subtropical Seashore Vegetation (Dayaram et al., 2019; Mucina & Rutherford, 2006). The conservation status of the Pondoland‐Ugu Sandstone Coastal Sourveld and Subtropical Seashore Vegetation are classified as ‘Vulnerable’, while Eastern Valley Bushveld, Scarp Forest, Subtropical Coastal Lagoons are classified as ‘Least threatened’ (Dayaram et al., 2019; Jewitt, 2018; Skowno et al., 2019).

Silaka and Hluleka Nature Reserves: Both nature reserves are located on the coast and are approximately 30 km apart. The vegetation types occurring in Silaka and Hluleka nature reserves are similar as described by Mucina and Rutherford (2006). Approximately 56% of each nature reserve is dominated by Transkei Coastal Belt and Scarp Forest vegetation is also found in the reserve (Dayaram et al., 2019; Mucina & Rutherford, 2006). The conservation status of Scarp Forest and Transkei Coastal Belt vegetation is classified as ‘Least threatened’ (Dayaram et al., 2019; Skowno et al., 2019).

Dwesa Nature Reserve: The vegetation of the area is dominated by Scarp Forest which covers approximately 62% of the reserve (Dayaram et al., 2019; Skowno et al., 2019). Other vegetation types are Subtropical Coastal Lagoons, Subtropical Dune Thicket, Subtropical Estuarine Salt Marshes, Subtropical Seashore Vegetation and Transkei Coastal Belt (Dayaram et al., 2019; Mucina & Rutherford, 2006). The conservation status of Scarp Forest, Transkei Coastal Belt vegetation, Subtropical Coastal Lagoons, Subtropical Estuarine Salt Marshes, Subtropical Seashore Vegetation and Subtropical Dune Thicket are all classified as ‘Least threatened’ (Dayaram et al., 2019; Skowno et al., 2019).

The climate of the study areas is classified as mild subtropical with relatively high humidity in the Wild Coast region of the Eastern Cape (Mucina & Rutherford, 2006). The climate conditions show similarity along the Wild Coast nature reserves, with the average temperature reaching 29°C on hot summer days and 9°C on cold winter days. Approximately 1000 mm of rain falls along the coastal belt annually, predominantly in the austral spring and autumn seasons (https://www.meteoblue.com/en/weather/forecast/modelclimate). There is no clear seasonality, probably because of the oceanic influences on the climate. As a result, based on the temperature and precipitation patterns, two periods become distinct: The cold period is represented by 6 months (April–September) of low temperature (4–15°C) and low precipitation (22–64 mm), while the warm period consists of 6 months (October–March) of relatively high temperatures (15–30°C) with the precipitation ranging from 70 to 110 mm.

### 2.2 Data collection

We used timed species counts (TSCs); a method outlined by Nalwanga et al. (2012). The timed species counts consist of a simple list of birds in which all species identified are recorded in the order in which they are encountered over a period of observation (Freeman et al., 2003). This method allows the observer to move around the landscape during the observation period (Nalwanga et al., 2012), and allows a bigger area to be covered (Bibby et al., 2000; Davies, 2002).

The timed species count approach was combined with an adaptive drive‐by survey (with a speed of 0–10 km/h; after Milton & Dean, 1998; Rahlao et al., 2010), to cover large areas within the reserves. The birds spotted during the drive‐by survey were also added to the main list of species. Surveys were not conducted on rainy and windy days.

We conducted the bird surveys at sampling locations during two foraging activities at peak times of the day (see the method in Mokotjomela et al., 2013a, 2013b): from 06:00 to 10:00 h (mornings) and 15:00 to 18:00 h (afternoons) for a period of 4 years (2017–2020). Sampling locations were systematically chosen in different vegetation types per nature reserve to achieve balanced observations in each site. Sampling was conducted during warm (i.e., December, January and February) and cold periods (i.e., June, July and August), since seasonality is not very clear in terms of temperatures.

On arrival at each location of the count, we recorded all bird species seen and heard calling during 10 min. After this, a scan sampling was conducted at 10‐min. intervals using binoculars (Bushnell: 10X42 Mg) and a spotting scope (SPEKTIV: 20X–60X). We used a digital camera (Canon Camera fitted with Sigma lens: 150–500 mm) to capture photographs of different bird species for further identification. The photograph of unknown bird species was taken, identified and recorded in the field notebook. The identification of the bird species was done using both calls and visuals and supported by different bird field guides (e.g. Sinclair et al., 2020, 2011) and online information (https://www.birdlife.org.za).

The recorded bird species were classified based on family, and adult body mass categories namely large birds (>150 g), medium‐size birds (50–150 g), small birds (30–49 g) and tiny birds (<30 g) (following Dennis & Westcott, 2007; Mokotjomela et al., 2013a). The birds' potential ecological roles were derived from their feeding mode and body mass categories (i.e. proxy of ecological role; following Hockey et al., 2005), and habitat associations (Hockey et al., 2005; Oatley, 1989). We sought to determine how these birds influence plants' reproductive mutualisms (i.e., nectarivores/pollinators, seed dispersers, insectivores and predators; following Hockey et al., 2005). Although different feeding modes and habitat preferences were not exclusive, we looked for a predominant one for each bird species as a basis for our classifications in this study (Hockey et al., 2005). Similarly, the recorded bird species were classified according to habitat preference using vegetation as the main determinant and the description of the main habitat, following Hockey et al. (2005). The bird size groups were divided according to adult body mass obtained from Hockey et al. (2005), and the developed size groups followed the criterion outlined in Mokotjomela (2012).

Climatic data (i.e., ambient temperature) were obtained for the four years of bird observations (i.e., 2017–2020 from the Google Earth Engine platform: Mutanga & Kumar, 2019) and were plotted to discern the linear variation during observation years. For temperature data, we used MODIS imagery‐ MOD11A2 V6 that provides an average of 8‐day land surface temperature (LST) with a 1‐km spatial resolution in a 1200 × 1200 km grid (Wan, 1999). The area of interest was defined as the nature reserves where the study was done. All nature reserves had similar coastal climatic patterns, but we decided to use Mkhambati Nature Reserve for benchmarking since it was the largest nature reserve. Queenstown was selected to represent inland climatic conditions due to its topography in order to allow a comparison of the coastal and inland conditions.

To determine the potential impact of habitat change on the diversity of bird species, the Normalised Difference Vegetation Index (NDVI) imagery set was used to classify the Landsat satellite pattern from the Google Earth Engine platform. A freely atmospherically corrected surface reflectance derived from the data produced by the Landsat 8 OLI/TIRS imagery available in GEE [Tier 1 products, ee.ImageCollection(‘LANDSAT/LC08/C02/T1_L2’)] was applied in this study. These images were collected from 1 January 2017 to 31 December 2020 (i.e., during years of bird observations). These products were atmospherically corrected using the Landsat‐8 Surface Reflectance Code (LaSRC) and contain cloud, shadow, water and snow masks, which were generated using the C Function of Mask (CFMask) algorithm (Amani et al., 2019; Foga et al., 2017). These images also contained five visible and near‐infrared (VNIR) bands and two short‐wave infrared (SWIR) bands processed to orthorectified surface reflectance, and one thermal infrared (TIR) band processed to orthorectified surface temperature. We used the spectral bands of the VNIR channel with a 30 m spatial resolution method to display the healthiness and greenness (relative biomass) of the vegetation (Prabhakara et al., 2015) by measuring the state of the plant's health based on the plant's reflection of light at certain frequencies (see Vukeya et al., 2023). The Landsat 8 sensor reflects both the near‐infrared spectrum in band 5 and visible red (RED) in band 4. The NDVI value ranges between −1.0 and +1.0 and reflects the healthiness of plants (greens). A low NDVI value of 0.1 and below corresponds with rocky and sandy areas or water (i.e., an NDVI below zero means no vegetation).

### 2.3 Statistical analyses

Frequency data were generated from records of bird species obtained from the field. The bird species counts data were analysed using a Generalised Linear Model (GLM) with Poisson error distribution and log link in SPSS software, version 20. First, a GLM was run to assess the number of bird species as predicted by habitat preference: the number of bird species was specified as the response variable, while the main habitat (vegetation) was treated as the predictor variable. The second GLM was applied to assess the number of bird species across feeding modes and body size classes. The number of bird species and families were specified as the response variable, while the bird body‐size class, different nature reserves and time (i.e. years of sampling and the seasons) were specified as predictor variables, with the feeding modes nested within the predictor variables.

We calculated the Shannon–Wiener Diversity Index (SWDI) to investigate overall bird species diversity and compare if the studied nature reserves and the observation years were different. We determined the functional diversity and compared its different components [i.e., richness (FRic), divergence (FDiv) and evenness (FEve)] across the nature reserves and the values were generated using the Fundiversity package in *R* software (Grenié & Gruson, 2023). Functional Richness (FRic), divergence (FDiv) and evenness (FEve) and values range from 0 to 1 (Schleuter et al., 2010). FRic is positively correlated to species diversity and FDiv while FEve is inversely related to the two indices (Mason et al., 2005).

We compared the number of bird species for preference of different habitats, as outlined in Hockey et al. (2005), and determined the most common bird species and their total frequency across the nature reserves over four years of observations. Using the General Linear Model with nested Analysis of Variance, the four nature reserves were specified as main groups and different vegetation types (i.e. habitat) were nested subgroups in SPSS software, version 20. We plotted the average monthly temperatures for the whole period of bird observations (2017–2020). We displayed data for the years 2018/2019 as unique years with the highest numbers of birds, to discern the potential influence of climatic conditions on the results.

To analyse the NDVI trends during the bird observation years (2017–2020), the change in the vegetation cover distribution per month in each of the four nature reserves was determined and presented using the Harmonic Model. The vegetation cover data for 2017 was used as the baseline to quantify the potential vegetation cover change. We also correlated the NDVI values for each nature reserve to the number of bird species records using the Spearman Rank Order Correlations.

There were no balanced replications of bird observation points within each vegetation type because of the dissimilarity of vegetation types and their composition in each nature reserve (i.e., Silaka and Hluleka Nature Reserves had only two types while Mkhambati had four types), and the difficult access of the land. However, we used the observation dates nested in each nature reserve as the replicates. To compare the distribution of the functional traits (i.e., different bird sizes and respective feeding modes) across the nature reserves, we performed Multiple Correspondence Analysis (MCA). The bird sizes were classified into different categories, namely large birds, medium birds, small birds and tiny birds (Mokotjomela, 2012), and their ecological functions of the birds derived from the feeding mode and main diet being pollinators (nectar), dispersers (fruits and seeds), predators (other birds and fruits) and carrion (scavengers) birds (Hockey et al., 2005) The nature reserves were used as supplementary data and were not contributing to the MCA components. We performed the MCA using the burt method on R statistics using the R packages FactoMiner and factoextra. Correlation coefficients range between ‐1 and +1. Larger negative and positive values also represent a strong correlation.

## 3 RESULTS

### 3.1 Comparing numbers of bird species in different nature reserves: Annual and seasonal patterns

Over a period of 864 hours of field sampling from 2017 to 2020, we accumulated 818 bird records containing 178 different bird species (i.e., 27% of known bird species in the Eastern Cape Province; *N* = 665) classified into 58 families. Silaka Nature Reserve had significantly more diverse bird families than Mkhambati Nature Reserve (Wald χ^2^ = 11.14; df = 3; *p* = .011), while other nature reserves were not significantly different (*p* > .05). The observation years were not significantly different in terms of bird families (Wald χ^2^ = 0.45; df = 3; *p* = .931).

Shannon–Wiener Diversity Indices showed very high overall species diversity and across the nature reserves (*H > 3.5*; Table 2) with all nature reserves being completely even (Table 2). Also, the overall annual bird species diversity across the observation years was very high (*H > 3.5*; Table 2).

**TABLE 2:** Shannon–Wiener diversity indices comparing the bird species diversity across the four nature reserves and the observation years.

Total species diversity		Species diversity in the nature reserves			
		DNR	HNR	MNR	SNR
*H*	4.8	4.5	4.4	4.4	4.2
*H*_*max*_	5.2	4.7	4.6	4.6	4.4
*Equitability*	0.9	1.0	1.0	1.0	1.0

Total species diversity		Observation years			
		2017	2018	2019	2020
*H*	3.6	4.2	3.9	3.8	4.2
*H*_*max*_	4.4	4.4	4.6	4.9	4.3
*Equitability*	0.5	1.0	0.8	0.8	1.0

		Functional diversity			
		DNR	HNR	MNR	SNR
*FRic*		1	0.999	1	1
*FDiv*		0.835	0.808	0.783	0.824
*FEve*		0.432	0.464	0.446	0.469

*Note*: Functional diversity values were generated using ‘Fundiversity’ package in *R* software. Components: Functional richness (FRic), Functional divergence (FDiv) and Functional evenness (FEve).

Abbreviations: DNR, Dwesa Nature Reserve; HNR, Hluleka Nature Reserve; MNR, Mkhambati Nature Reserve; SNR, Silaka Nature Reserve.

Overall, the nature reserves displayed high functional richness (FRic = 1); high functional divergence (FDiv = 0.8) and moderate functional evenness (FEve = 0.4; Table 2).

There was no significant difference in the number of bird species among the nature reserves (Wald χ^2^ = 4.11; df = 3; *p* = .249; Table 3). Out of 178 bird species, we noted that 32 bird species representing 30 families were recorded in all four nature reserves (Table S1). Overall, the forest bird species were more dominant (42.1%; *N* = 178) throughout the observations (Wald χ^2^ = 49.3; df = 7; *p* < .001; Table 3) than other habitat species. The nested subsets analyses also showed significantly greater numbers of forest bird species across the nature reserves [*F*_(9,648)_ = 2.9; *p =* .002] suggesting the forest habitat accounts for a large amount of observed variation.

**TABLE 3:** Generalised Linear Models' (GLM) significant differences among the bird species' habitat preference (Hockey et al., 2005).

Parameter	*B*	Std. error	Hypothesis test		
			Wald chi‐square	df	*p*‐Value
*Habitat preference*					
**Overall differences—Test model effect**			**47.3**	**7**	**.000**
Generalist	0.441	0.3445	1.636	1	.201
2 Coastal	0.607	0.5306	1.309	1	.253
3 Desert	0.196	0.5005	0.153	1	.696
4 **Forest**	**0.710**	**0.2595**	**7.475**	**1**	**.006**
5 Fynbos	0.377	0.5060	0.555	1	.456
6 **Grassland**	**1.088**	**0.4098**	**7.044**	**1**	**.008**
7 Savanna	0.435	0.2971	2.146	1	.143
8 Wetland	0^a^				

^a^ A parameter that was selected as reference for comparison of the significance.

Bold indicates statistical significant value (p < .05).

**FIGURE 2:**
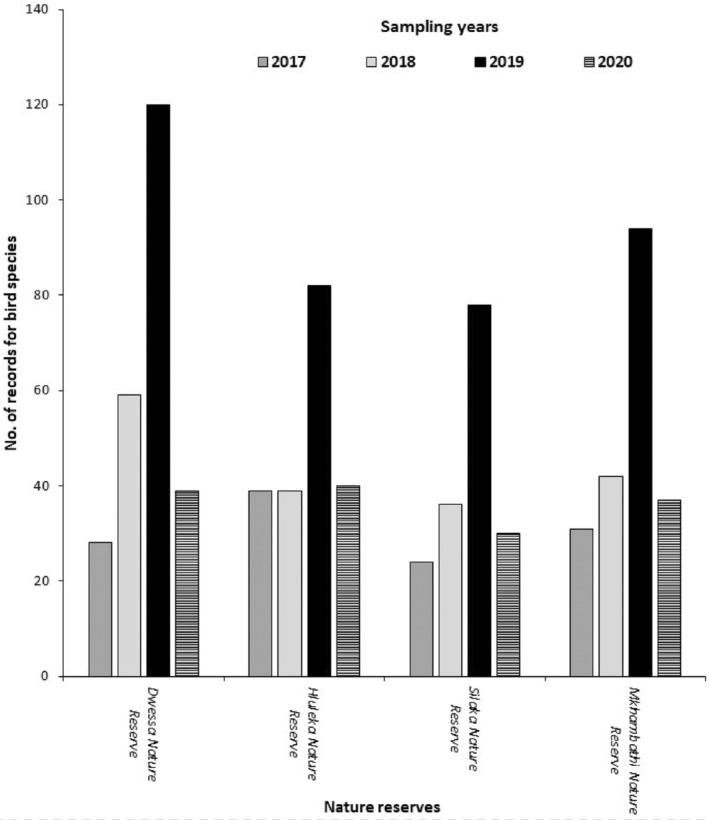
Annual differences in the total number of records of different bird species observed in four different nature reserves for 4 years.

There were no significant differences in the numbers of species across the years (Pearson χ^2^ = 453.; df = 535; *p* = .989). However, the numbers of birds differed significantly among the sampling years, with 2019 showing the largest number of birds across all nature reserves (Wald χ^2^ = 6.13; df = 1; *p* = .013; Figure 2). The warm and cold periods were not significantly different in the number of bird species (Wald χ^2^ = 0.79; df = 1; *p* =.375).

### 3.2 Ecological roles provided by birds in different nature reserves and different observation years

**FIGURE 3:**
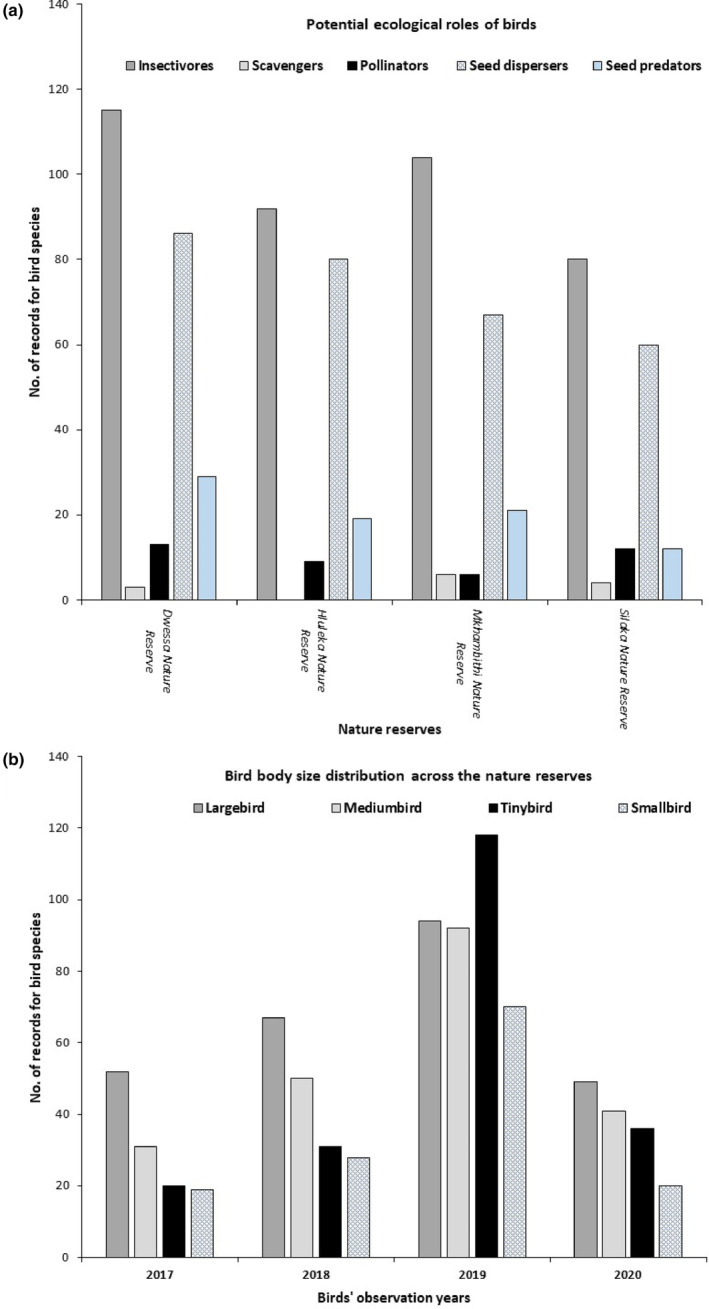
Number of different bird species having relevant ecological roles in vegetation maintenance, including other birds having indirect roles across the different nature reserves (a); the bird body mass class distribution during the different years of sampling (b).

Overall, there were significant differences in the numbers of birds among different feeding mode classifications, which is a species function's proxy (Wald χ^2^ = 18.31; df = 3; *p* < .001: Figure 3a and Table 4). Seed dispersing birds, scavengers and insectivores had a significantly greater number of records than seed predators and pollinators, while the pollinator birds and seed predators were not significantly different in number (*p* > .05; Table 4).

**TABLE 4:** Generalised Linear Models' (GLM) significant differences among the bird species' functions based on feeding mode, and the body‐size classes' distribution during different years of observations.

Parameter	*B*	Std. error	Hypothesis test		
			Wald chi‐square	df	*p*‐Value
*Brid function/role*					
**Overall differences—Test model effects**			**18.31**	**3**	**.000**
**Insectivores**	**0.592**	**0.1307**	**20.49**	**1**	**.000**
**Scavengers**	**1.213**	**0.3873**	**9.81**	**1**	**.002**
**Seed dispersers**	**1.197**	**0.1341**	**79.58**	**1**	**.000**
Pollinators	0.202	0.2127	0.91	1	.341
Seed predators	0^a^	–	–	–	–
*Body size class*					
**Overall differences—Test model effects**			**109.49**	**4**	**.000**
Large bird (>150 g)	−0.204	0.1081	3.55	1	.060
Medium‐size bird (50‐149 g)	−0.013	0.1054	0.02	1	.898
**Small bird (30‐49 g)**	**−0.436**	**0.1180**	**13.63**	**1**	**.000**
Tiny bird (<30 g)	0^a^	–	–	–	–

^a^ A parameter that was selected as reference for comparison of the significance.

Bold indicates statistical significant value (p < .05).

The numbers of birds in the small body‐size class were significantly fewer than large birds (Wald χ^2^ = 109.49; df = 4; *p* < .001: Table 4), while all bird body‐size classes were not significantly different (Figure 3b and Table 4).

### 3.3 The role of vegetation cover on bird diversity

**FIGURE 4:**
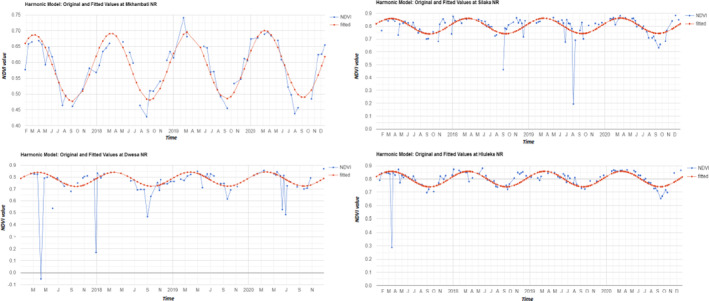
Normalised Difference Vegetation Index (NDVI) was used to determine the role of vegetation cover on observed bird species diversity over 2017–2020 in Mkhambati (a), Silaka (b), Dwesa (c) and Hluleka (d) Nature Reserves, Eastern Cape. To depict the average NDVI values' change, the Harmonic model was fitted.

We found a non‐significant correlation between the number of bird species and vegetation cover (i.e., NDVI; for all Spearman correlations coefficients, *p* > .05) across the four nature reserves during the bird observation years suggesting vegetation cover did not influence the numbers of bird species observed (Figure 4). The four nature reserves showed relatively consistent NDVI indices across the years although Mkhambati Nature Reserve showed bigger oscillations than the other nature reserves possibly due to the dominance of grassland vegetation in which summer and winter are substantially different (Figure 4A, B, C & D).

### 3.4 Multi correspondence analysis: Birds' body sizes and feeding modes across different nature reserves

**FIGURE 5:**
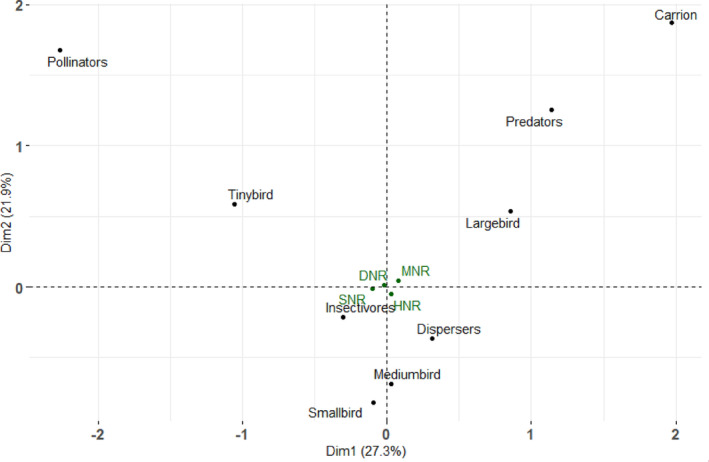
Multiple Correspondence Analysis (MCA) showing the relationship between the bird sizes (i.e. proxi for dispersal distance ability), and functions (i.e., based on the feeding modes) of birds in the ECPTA coastal nature reserves. DNR, Dwesa Nature Reserve; HNR, Hluleka Nature Reserve; MNR, Mkhambati Nature Reserve; SNR, Silaka Nature Reserve.

The first dimension of the multi correspondence analysis (MCA) accounts for 27.3% of the explained variance and explained most of the variation in the data, and dimensions two and three make up for 21.9% and 15.2% of the explained variance, respectively (Figure 5). All nature reserves had similar distribution of the study functional traits in the study. All three dimensions account for 64.4% of variation in the original data (Table S2). Large birds and predators show strong negative correlation with dimension 1 of the MCA, while pollinators, and tiny birds have a strong positive correlation with dimension 1 (Table S2).

In the second dimension, only medium birds show a strong negative correlation with this dimension, and tiny birds, carrion, pollinators and predators have a strong positive correlation with the dimension. In the third dimension, only carrions have a strong positive correlation with this dimension, and predators show a strong negative correlation (Table S3). Upon comparing the association between bird sizes and their functions in the ecosystem, large birds are mainly associated with predation and carrion which provide indirect long dispersal distances (Figure 5). Tiny birds that are mainly insectivores were mostly associated with pollination services. Small birds and medium birds had similar composition of species in terms of functionality and served as the dispersers and pollinators through insectivory while in large and tiny birds, each category has its distinct functionality across the continuum (Figure 5).

## 4 DISCUSSION

Birds provide important ecosystem services for both the natural and built environments (Şekercioğlu et al., 2016; Whelan et al., 2008), and consequently, knowledge of species diversity could be used to predict the potential ecological functions in local habitat maintenance (Mao et al., 2023; Morante‐Filho & Faria, 2017). In this study, we report bird species' diversity and the potential roles of these birds for habitat maintenance in four nature reserves located in the east wildcoast of South Africa.

Our findings show that the four nature reserves contained 178 (27%; *N* = 665) of the whole Eastern Cape Province's species pool, partly explained by high micro‐habitat heterogeneity in each nature reserve (Leaver et al., 2019; Schütz & Schulze, 2015), since there were significant correlation between the NDVI and numbers of birds records. The wood harvesting and invasion by alien plant species may partly create a favourable habitat mosaic for birds in the Eastern Cape Province (Leaver & Cherry, 2020), which partly could explain our results of relatively large numbers of bird species. This is a relatively high species diversity given the small spatial coverage of the four nature reserves that were sampled during the study, and it was matched by high functional richness which is reportedly positively correlated to the number of species (Li et al., 2022; Schleuter et al., 2010). However, Li et al. (2022) reported an inverse relationship between species diversity and functional evenness and therefore, the moderate functional evenness across the nature reserves suggests stability of the biodiversity of the nature reserves since the high functional diversity increases habitat resilience in protected areas (Cottee‐Jones et al., 2015; Lotz, 2021). Furthermore, our moderate functional evenness indices indicate a balanced utilisation of resources and thus productivity (Mason et al., 2005). Our results are consistent with reports that diversity in environmental conditions, especially in vegetation types, can increase the diversity of bird communities (BirdLife South Africa, 2022; Mao et al., 2023), and a similar observation was reported in central Argentina (Leveau, 2019; Tilman et al., 1997).

The absence of significant differences in the numbers of bird species among the nature reserves may be a result of the presence of forest vegetation components across the nature reserves—the Maputaland–Pondoland–Albany hotspot for biodiversity (Berliner, 2009). Forest habitats tend to provide more resources than other vegetation types in the Eastern Cape province in South Africa (Bitani et al., 2020; Leaver & Cherry, 2020; Maseko et al., 2020; Oatley, 1989). Similarly, our finding of a relatively large number of different forest bird species may be attributed to those abundant resources although there are no predictable fruit patterns in forest patches (Gumede et al., 2022; Hart et al., 2013) of which may influence bird movement between the forest patches, and thus, associated dispersal services (Gumede et al., 2022; Mao et al., 2023; Maseko et al., 2020). Furthermore, the protected biodiversity conservation areas experience minimal anthropogenic habitat disturbance and thus adequate structural resources, thereby providing a safe living place for certain bird species (Evans et al., 2006; Gomes et al., 2008; Gregory et al., 2003; Lecina‐Diaz et al., 2019; Maseko et al., 2020).

We expected Mkhambati Nature Reserve to have the highest number of bird species since it is an Important Birding Area (IBA) in South Africa (BirdLife South Africa, 2022), and because of its relatively large size, which reportedly has a positive effect on species diversity (Ehlers‐Smith et al., 2018; Evans et al., 2006). However, it is possible that difficult access to the other parts of the area might have limited the bird sightings. Nevertheless, Evans et al. (2006) reported that the diversity of birds did not correlate with the size of the protected area in South Africa, suggesting that the small areas also contribute effectively to the maintenance of the bird species' richness.

We also found that Silaka Nature Reserve had significantly larger numbers of the birds' families than other nature reserves, despite the nature reserve's relatively small size, which could possibly be attributed to its location adjacent to the town of Port St Johns. Due to limited development, Port St Johns may represent intermediate disturbances that diversify the local habitat thereby leading to high bird species' diversity (Blair, 1999). Urban gardens increase the local habitat diversity and tend to offer resources for many birds (Mokotjomela, 2012; Shochat et al., 2010) because the vegetation is usually dominated by fruiting woody alien species (Daniels & Kirkpatrick, 2006; Mokotjomela et al., 2013a, 2022; Shochat et al., 2010). The results of this study concur with the recent studies suggesting the existence of high bird species' diversity, requiring conservation efforts in the Eastern Cape forests (e.g. Leaver, 2020; Mulvaney, 2021).

Usually, birds display seasonal diet and behavioural shifts elicited by environmental conditions (Herrera, 1984; Karr, 1976; Kearney & Porter, 2009; Vukeya et al., 2022; Williams, 2006), with many and diverse bird species observed more frequently in warm than in cold days (Aikins et al., 2019; O'Connor & Hicks, 1980; Walther et al., 2017). Similarly, high bird species diversity in South Africa is associated with warm and rainy growing seasons because of the abundant availability of resources (see, Aikins et al., 2019; Karr, 1976), and breeding season in eastern coastal forests in South Africa (Ehlers‐Smith et al., 2018). However, we found that bird species' diversity was not affected by seasonal variations (i.e. cold and warm seasons) in the study areas, suggesting constant and liveable environmental conditions. This finding is consistent with Karr (1976) who reported that seasonal variation in avian community structure decreases with increasing vegetation complexity—indeed, the Eastern Cape Wild Coast preserves complex and threatened vegetation patches (Mucina & Rutherford, 2006; Skowno et al., 2019). The coastal areas have climates that are more humid than inland climates in the Eastern Cape Province (Nkamisa et al., 2022; Landman et al., 2017), which has positive effects on plant‐based food recourses as well as the invertebrates (Karr, 1976): all of these may attract inland birds during periods of drought and food scarcity. Consistently, we found significantly higher records of bird species in 2018/2019 than in other years, which is partly because of a combination of observed extreme drought conditions and arrival of the migrant bird species. The exposure of birds to high temperatures can incur high water and energy costs, which can ultimately result in severe fitness costs and lethal hyperthermia (McKechnie & Wolf, 2010; Welbergen et al., 2008). It is possible that the humid coastal environmental conditions provided buffer refugia against the extreme inland temperatures for inland bird species during 2018/2019; these conditions have previously been reported to result in localised and landscape‐dependent migrations of birds (Hockey et al., 2005; Mokotjomela, 2012). Furthermore, the complex vegetation cover of the Eastern Cape coastal areas must have increased buffering of extreme environmental conditions such as drought (Karr, 1976), and thus attracted the migrant birds for overwintering purposes as reported elsewhere in Free State (Vukeya et al., 2022). In addition, because birds are capable of tracking vegetation‐based food resources such as fleshy fruits and nectar (Mokotjomela et al., 2013b; Saracco et al., 2004; Telleria et al., 2008), the inland bird populations could have moved from their usual habitats to the humid coastal areas.

The Eastern Cape Wild Coast is renowned worldwide for its high plant endemism (Skowno et al., 2019). Because birds play a critical role in habitat maintenance, functioning and vegetation regeneration (Lotz, 2021; Morante‐Filho & Faria, 2017; Pimm, 1986; Sekercioglu, 2006; Wenny et al., 2011; Whelan et al., 2015), understanding how birds functional traits influence plants' vegetation dynamics in protected biodiversity areas is important. The Multiple Correspondence Analysis (MCA) demonstrated a positive correlation between the bird sizes and the bird functions in the nature reserves with large birds mainly associated with predators and carrions of which may provide indirect and secondary long‐distance seed dispersal as observed in Mkhambati Nature Reserve. For example, large birds preying (e.g. carrion in this study) on small disperser birds have been reported and has positive conservation implications for plants with restricted home ranges (Corlett, 2017). The finding that most of the bird species recorded in this study were potential seed dispersers (35.8%; *N* = 818) suggests that nature reserves could directly benefit effective seed dispersal as an important process in plant recruitment (Schurr et al., 2009; Wang & Smith, 2002). This finding is consistent with a recent study showing that forest generalist bird species are relatively abundant and persist in the fragmented forest thereby guaranteed seed dispersal services in Indian Ocean Coastal Belt Forests, South Africa (Bitani et al., 2020). Our finding that small birds and medium birds had a similar composition of species in terms of functionality and served as the dispersers are consistent with previous studies emphasising their importance in vegetation dynamics (Godinez‐Alvarez et al., 2020; Mao et al., 2023; Silva et al., 2020). The importance of birds in improving seed germination of native plant species has been well studied in South Africa (Mokotjomela, 2012; Vukeya et al., 2021, 2023), and we suggest that birds promote coastal plant population persistence in the Eastern Cape Province. The non‐significantly different numbers of birds in large‐ and medium‐size body mass classes may suggest the complementary seed dispersal services in terms of dispersal distances (Godinez‐Alvarez et al., 2020; Mokotjomela et al., 2015, 2021; Schurr et al., 2009). Whereas seed predator birds such as doves could reduce density‐based competition for the dispersed seeds (Mokotjomela et al., 2015), they have also been shown to have a positive effect on seed germination through scarification of the few seeds surviving gut passage (Mokotjomela et al., 2015). Finally, the occurrence of different vegetation specialist bird species may indicate a high potential for diverse movements between various patches, which may guarantee the spread of seeds.

It is important to note that the lower number of pollinators in the recorded bird species is not a true reflection of available pollination services since some avian disperser species also occasionally feed on nectar for diet variation (Hockey et al., 2005; Mokotjomela, 2012; Mokotjomela et al., 2015). We contend that the significant effect that insectivores and carrions displayed in the MCA plots, suggest the potential availability of indirect pollination services when, for example, they are foraging either for insects or any diet that might have pollen, since reports show that each plant species may be dependent on both ‘legitimate’ and ‘non‐legitimate’ mutualist partners (Johnson et al., 2009; Mokotjomela et al., 2015). We also found that the tiny birds that are mainly insectivores were mostly associated with pollinator services probably due to either specialised architecture of their beaks or the ability to visit flowers easily with their small bodies especially in Dwesa Natur Reserve. South Africa, like Australia, has a high percentage of flora that is adapted for pollination by either birds or mammals, and indeed, bird pollination systems can be complex (Johnson et al., 2009). Moreover, many plant species in grasslands, a common vegetation unit in the study sites, are pollinated by insects in South Africa (Johnson et al., 2009; Skowno et al., 2019). Indeed, the apparent constant presence of birds in the study sites throughout the sampling years could perpetuate the provision of ecological services (Ehlers‐Smith et al., 2018), and improve habitat productivity to maintain the high plant endemism that is protected in the Maputaland–Pondoland–Albany region (Fang et al., 2012; Skowno et al., 2019; Tilman et al., 1997). Our results are consistent with reports that biodiversity conservation generally aims to maintain taxonomic species richness, as well as functional richness to sustain the overall habitat functioning of food webs to elicit habitat resilience and stability (Lotz, 2021; Pecl et al., 2017).

## AUTHOR CONTRIBUTIONS

**Thabiso M Mokotjomela:** Conceptualization (equal); data collection and curation (equal); formal analysis (equal); methodology (equal); visualization (equal); writing – original draft (equal); writing – review and editing (equal). **Loyd Rodney Vukeya:** Conceptualization (equal); data curation (equal); formal analysis (equal); methodology (equal); visualization (equal); writing – original draft (equal); writing – review and editing (equal). **Lwandiso Pamla:** Conceptualization (equal); data curation (equal); investigation (equal); methodology (equal); project administration (equal); visualization (equal); writing – original draft (equal); writing – review and editing (equal). **Zimbini Scott:** Conceptualization (equal); formal analysis (equal); methodology (equal); software (equal); writing – review and editing (equal).

## FUNDING INFORMATION

All information was provided no specific grant number was awarded for the study.

## CONFLICT OF INTEREST STATEMENT

The authors declare that no conflict of interest exist.

### OPEN RESEARCH BADGES

This article has earned an Open Data badge for making publicly available the digitally‐shareable data necessary to reproduce the reported results. The data is available at https://doi.org/10.5061/dryad.p5hqbzkrp.

## Supporting information


Table S1:

## Data Availability

Mokotjomela et al. (2022), Wild coast birds 2017‐2022_Jun 2022_DRYAD, Dryad, Dataset, https://doi.org/10.5061/dryad.p5hqbzkrp.
