# Supplementary material for: The critical role of coastal protected areas in buffering impacts of extreme climatic conditions on bird diversity and their ecosystem services' provisioning in the Eastern Cape Province, South Africa
Source: Ecol Evol. 2023 Oct 20;13(10):e10452. doi: 10.1002/ece3.10452 (PMC10587736; doi:10.1002/ece3.10452)
Supplement: Supplementary file 1 — Table S1: [file ECE3-13-e10452-s001.docx]

**Supplementary material:**

**Table S1**: List of bird species and families recorded in the four nature reserves. Bird descriptions/attributes (i.e., body mass), potential role in vegetation maintenance, and habitat preferences (defined by vegetation) were obtained from Hockey et al. (2005), and observation frequency. IUCN conservation status: LC = Least concern, VU = Vulnerable, NE = Near endangered, NT = Near threatened, E = Endangered; Ecological roles: Scav = Scavengers, Pred = Seed predators, Disp = Seed dispersers, Poll = Pollinators, and Ins = Insectivore bird species; Species presence in each nature reserve is marked with ⚫ and absence x.

|  | **Names** |  | **Species attributes** | | | | | **Reserve name (Bird Presence/Absence)** | | | |
| --- | --- | --- | --- | --- | --- | --- | --- | --- | --- | --- | --- |
| ID | **Latin name** | **Family** | **Body mass (g)** | **IUCN Status** | **Behaviour** | **Habitat** | **Ecological role** | **DNR** | **HNR** | **MNR** | **SNR** |
| 1 | *Accipiter badius* | Accipitridae | 141 | LC | resident and some local movement | forest | Scav | **x** | **x** | **x** | **⚫** |
| 2 | *Accipiter melanoleucus* | Accipitridae | 980 | LC | resident | forest | Pred | **x** | **x** | **⚫** | **x** |
| 3 | *Accipiter tachiro* | Accipitridae | 510 | LC | resident | forest | Pred | **⚫** |  | **⚫** | **⚫** |
| 4 | *Acrocephalus palustris* | Sylviidae | 11 | LC | resident and altitudinal migrant | wetland | Ins | **x** | **⚫** | **x** | **⚫** |
| 5 | *Alcedo semitorquata* | Alcedinidae | 40 | LC | resident and altitudinal migrant | wetland | Pred | **x** | **x** | **⚫** | **x** |
| 6 | *Alopochen aegyptiaca* | Anatidae | 1990 | LC | resident and nomad | wetland | Ins | **⚫** | **x** | **x** | **x** |
| 7 | *Amblyospiza albifrons* | Ploceidae | 57 | LC | some local movement | wetland | Disp | **⚫** | **⚫** | **x** | **⚫** |
| 8 | *Anas undulata* | Anatidae | 502 | LC | resident and nomad | wetland | Disp | **⚫** | **⚫** | **x** | **x** |
| 9 | *Andropadus importunus* | Pycnonotidae | 36 | LC | resident | forest | Disp | **⚫** | **⚫** | **x** | **⚫** |
| 10 | *Anhinga rufa* | Anhingidae | 1350 | LC | resident, nomad and some local movement | wetland | Pred | **⚫** | **⚫** | **x** | **x** |
| 11 | *Anthobaphes violacea* | Nectariniidae | 26 | LC | resident and some local movement | fynbos | Poll | **x** | **x** | **x** | **⚫** |
| 12 | *Anthus cinnamomeus* | Motacillidae | 30 | LC | resident and altitudinal migrant | grass | Ins | **⚫** | **x** | **x** | **x** |
| 13 | *Apalis thoracica* | Accipitridae | 510 | LC | resident | forest | Pred | **x** | **x** | **x** | **⚫** |
| 14 | *Apalis thoracica* | Cisticolidae | 14 | LC | resident and some local movement | forest | Ins | **⚫** | **⚫** | **x** | **⚫** |
| 15 | *Apaloderma narina* | Trogonidae | 95 | LC | resident and some local movement | forest | Ins | **⚫** | **⚫** | **x** | **x** |
| 16 | *Apus apus* | Apodidae | 37 | LC | Intercontinental migrant | savanna | Ins | **⚫** | **x** | **⚫** | **x** |
| 17 | *Apus barbatus* | Apodidae | 42 | LC | resident and intra african migrant | generalist | Ins | **⚫** | **⚫** | **⚫** | **⚫** |
| 18 | *Apus caffer* | Apodidae | 24 | LC | intra african migrant | generalist | Ins | **⚫** | **⚫** | **⚫** |  |
| 19 | *Apus horus* | Apodidae | 26 | LC | resident and intra african migrant | generalist | Ins | **⚫** | **⚫** |  | **⚫** |
| 20 | *Ardea cinerea* | Ardeidae | 2000 | LC | resident and some local movement | wetland | Pred | **⚫** | **⚫** | **⚫** | **⚫** |
| 21 | *Batis capensis* | Malaconotidae | 13 | LC | resident and some local movement | forest | Ins | **⚫** | **⚫** | **x** | **⚫** |
| 22 | *Batis molitor* | Malaconotidae | 14 | LC | resident | savanna | Ins | **x** | **x** | **x** | **⚫** |
| 23 | *Bostrychia hagedash* | Threskiornithidae | 1238 | LC | resident and nomad | forest | Ins | **⚫** | **⚫** | **⚫** | **⚫** |
| 24 | *Bubo africanus* | Strigidae | 696 | LC | resident | savanna | Ins | **x** | **⚫** | **⚫** | **x** |
| 25 | *Bubulcus ibis* | Ardeidae | 220 | LC | resident and nomad | grass | Ins | **⚫** | **⚫** | **x** | **x** |
| 26 | *Bucorvus leadbeateri* | Bucorvidae | 3800 | VU | resident | savanna | Ins | **x** | **x** | **⚫** | **x** |
| 27 | *Buteo Buteo* | Accipitridae | 1400 | LC | resident and intercontinental migrant | grass | Pred | **x** | **⚫** | **x** | **x** |
| 28 | *Buteo rufofuscus* | Accipitridae | 1695 | LC | resident and some local movement | generalist | Pred | **⚫** | **⚫** | **⚫** | **⚫** |
| 29 | *Buteo trizonatus* | Accipitridae | 700 | NT | resident and some local movement | forest | Pred | **x** | **x** | **⚫** | **x** |
| 30 | *Bycanistes bucinator* | Bucerotidae | 670 | LC | resident and some local movement | forest | Disp | **⚫** | **⚫** | **⚫** | **⚫** |
| 31 | *Calandrella cinerea* | Alaudidae | 24 | LC | resident, nomad and some local movement | grass | Disp | **x** | **⚫** | **⚫** | **x** |
| 32 | *Camaroptera brachyura* | Cisticolidae | 11 | LC | resident and some local movement | forest | Ins | **x** | **⚫** | **x** | **⚫** |
| 33 | *Campethera notata* | Picidae | 70 | NT | resident | forest | Ins | **⚫** | **⚫** | **x** | **x** |
| 34 | *Campethera scriptoricauda* | Picidae | 70 | LC | resident | forest | Pred | **⚫** | **⚫** | **x** | **x** |
| 35 | *Caprimulgus europaeus* | Caprimulgidae | 85 | LC | Intercontinental migrant | savanna | Ins | **x** | **x** | **⚫** | **x** |
| 36 | *Ceblepyris caesius* | Campephagidae | 66 | LC | resident and intra african migrant | forest | Ins | **⚫** | **⚫** | **x** | **x** |
| 37 | *Cecropis abyssinica* | Hirundinidae | 21 | LC | resident and intra african migrant | grass | Ins | **⚫** | **x** | **⚫** | **x** |
| 38 | *Cecropis cucullata* | Hirundinidae | 35 | LC | resident | grass | Ins | **⚫** | **⚫** | **x** | **x** |
| 39 | *Centropus burchellii* | Centropodidae | 170 | NE | resident | wetland | Ins | **⚫** | **⚫** | **⚫** | **⚫** |
| 40 | *Centropus superciliosus* | Centropodidae | 150 | LC | resident and some local movement | wetland | Pred | **⚫** | **⚫** | **x** | **x** |
| 41 | *Ceryle rudis* | Cerylidae | 84 | LC | resident and some local movement | wetland | Pred | **⚫** | **⚫** | **⚫** | **⚫** |
| 42 | *Chalcomitra amethystina* | Nectariniidae | 14 | LC | resident, nomad and some local movement | savanna | Poll | **⚫** | **⚫** | **x** | **⚫** |
| 43 | *Chlorophoneus olivaceus* | Malaconotidae | 39 | LC | resident | forest | Ins | **x** | **x** | **x** | **⚫** |
| 44 | *Cinnyris afer* | Nectariniidae | 18 | LC | resident and some local movement | forest | Poll | **⚫** | **⚫** | **⚫** | **⚫** |
| 45 | *Cinnyris bifasciatus* | Nectariniidae | 11 | LC | resident, nomad and some local movement | forest | Poll | **⚫** | **x** | **x** | **x** |
| 46 | *Cinnyris chalybeus* | Nectariniidae | 8 | LC | resident and some local movement | fynbos | Poll | **⚫** | **⚫** | **⚫** | **⚫** |
| 47 | *Cisticola aberrans* | Cisticolidae | 13 | LC | resident and some local movement | forest | Ins | **x** | **x** | **⚫** | **x** |
| 48 | *Cisticola ayresii* | Cisticolidae | 11 | LC | resident and some local movement | grass | Ins | **⚫** | **x** | **x** | **x** |
| 49 | *Cisticola juncidis* | Sylviidae | 9 | LC | resident and intercontinental migrant | grass | Ins | **x** | **⚫** | **x** | **x** |
| 50 | *Colius striatus* | Collidae | 55 | LC | resident, some local movement and altitudinal migrant | forest | Disp | **⚫** | **⚫** | **⚫** | **⚫** |
| 51 | *Columba arquatrix* | Columbidae | 429 | LC | resident and some local movement | forest | Disp | **⚫** | **x** | **x** | **x** |
| 52 | *Columba delegorguei* | Columbidae | 170 | LC | resident | forest | Disp | **⚫** | **⚫** | **⚫** | **x** |
| 53 | *Columba guinea* | Columbidae | 344 | LC | resident and some local movement | desert | Disp | **x** | **x** | **⚫** | **x** |
| 54 | *Coracias garrulus* | Coraciidae | 122 | LC | Intercontinental migrant | savanna | Ins | **⚫** | **x** | **x** | **x** |
| 55 | *Corvus albicollis* | Corvidae | 762 | LC | resident | savanna | Ins | **⚫** | **⚫** | **⚫** | **⚫** |
| 56 | *Corvus albus* | Corvidae | 700 | LC | resident and intra african migrant | savanna | Ins | **x** | **x** | **⚫** | **⚫** |
| 57 | *Corvus capensis* | Corvidae | 537 | LC | resident | savanna | Ins | **x** | **x** | **⚫** | **x** |
| 58 | *Corythornis cristatus* | Alcedinidae | 17 | LC | resident and intra african migrant | wetland | Pred | **⚫** | **⚫** | **⚫** | **⚫** |
| 59 | *Cossypha dichroa* | Muscicapidae | 48 | LC | resident and altitudinal migrant | forest | Disp | **⚫** | **⚫** | **x** | **⚫** |
| 60 | *Coturnix coturnix* | Phasianidae | 96 | LC | intra african migrant | grass | Disp | **x** | **x** | **⚫** | **x** |
| 61 | *Crithagra flaviventris* | Fringillidae | 30 | LC | resident, nomad and some local movement | desert | Disp | **⚫** | **⚫** | **⚫** | **x** |
| 62 | *Crithagra gularis* | Fringillidae | 20 | LC | resident, some local movement and altitudinal migrant | forest | Disp | **x** | **⚫** | **x** | **x** |
| 63 | *Crithagra mozambica* | Fringillidae | 13 | LC | resident and intra african migrant | savanna | Disp | **⚫** | **⚫** | **⚫** | **⚫** |
| 64 | *Crithagra sulphurata* | Fringillidae | 30 | LC | resident and nomad | forest | Disp | **⚫** | **x** | **x** | **x** |
| 65 | *Cuculus canorus* | Cuculidae | 130 | LC | Intercontinental migrant | forest | Ins | **⚫** | **x** | **x** | **x** |
| 66 | *Cyanomitra veroxii* | Nectariniidae | 13 | LC | nomad and some local movement | forest | Ins | **⚫** | **x** | **x** | **x** |
| 67 | *Cypsiurus parvus* | Apodidae | 14 | LC | resident and some local movement | savanna | Ins | **x** | **x** | **⚫** | **x** |
| 68 | *Dendropicos fuscescens* | Picidae | 31 | LC | resident | savanna | Ins | **⚫** | **x** | **⚫** | **⚫** |
| 69 | *Dendropicos griseocephalus* | Picidae | 42 | LC | resident | forest | Ins | **⚫** | **⚫** | **x** | **⚫** |
| 70 | *Dessonornis caffer* | Muscicapidae | 28 | LC | resident and some local movement | forest | Disp | **⚫** | **⚫** | **⚫** | **⚫** |
| 71 | *Dicrurus adsimilis* | Dicururidae | 50 | LC | resident and some local movement | savanna | Ins | **⚫** | **x** | **⚫** | **⚫** |
| 72 | *Dicrurus ludwigii* | Dicururidae | 28 | LC | resident | forest | Ins | **⚫** | **x** | **⚫** | **⚫** |
| 73 | *Diomedea exulans* | Diomedeidae | 8130 | VU | Intercontinental migrant | Coastal | Scav | **⚫** | **x** | **x** | **x** |
| 74 | *Dryoscopus cubla* | Malaconotidae | 36 | LC | resident and nomad | forest | Ins | **⚫** | **⚫** | **x** | **⚫** |
| 75 | *Emberiza capensis* | Fringillidae | 21 | LC | resident and some local movement | fynbos | Disp | **⚫** | **x** | **x** | **x** |
| 76 | *Estrilda astrild* | Estrildidae | 9 | LC | resident and some local movement | grass | Disp | **x** | **⚫** | **⚫** | **x** |
| 77 | *Euplectes axillaris* | Ploceidae | 32 | LC | resident | grass | Disp | **⚫** | **⚫** | **⚫** | **x** |
| 78 | *Euplectes progne* | Ploceidae | 46 | LC | resident and some local movement | grass | Disp | **⚫** | **⚫** | **⚫** | **x** |
| 79 | *Falco concolor* | Falconidae | 210 | VU | vagrant | forest | Pred | **x** | **⚫** | **x** | **x** |
| 80 | *Falco peregrinus* | Falconidae | 1000 | LC | resident and some local movement | generalist | Pred | **x** | **x** | **⚫** | **x** |
| 81 | *Fraseria caerulescens* | Muscicapidae | 17 | LC | resident and intra african migrant | forest | Ins | **⚫** | **⚫** | **⚫** | **x** |
| 82 | *Gallirex porphyreolophus* | Musophagidae | 328 | LC | resident and some local movement | forest | Disp | **x** | **x** | **x** | **⚫** |
| 83 | *Gyps coprotheres* | Accipitridae | 8600 | EN | resident and some local movement | grass | Scav | **x** | **x** | **⚫** | **x** |
| 84 | *Haematopus moquini* | Haematopodidae | 692 | NT | resident and some local movement | Coastal | Ins | **⚫** | **x** | **x** | **x** |
| 85 | *Halcyon albiventris* | Dacelonidae | 60 | LC | resident and intra african migrant | forest | Ins | **⚫** | **⚫** | **x** | **⚫** |
| 86 | *Halcyon senegaloides* | Dacelonidae | 85 | LC | intra african migrant | forest | Pred | **⚫** | **x** | **x** | **x** |
| 87 | *Haliaeetus vocifer* | Accipitridae | 2780 | LC | resident and some local movement | wetland | Pred | **⚫** | **⚫** | **⚫** | **x** |
| 88 | *Hedydipna collaris* | Nectariniidae | 11 | LC | resident | forest | Disp | **⚫** | **⚫** | **x** | **⚫** |
| 89 | *Hemimacronyx chloris* | Motacillidae | 50 | VU | resident | grass | Ins | **x** | **x** | **⚫** | **x** |
| 90 | *Hirundo albigularis* | Hirundinidae | 21 | LC | intra african migrant | wetland | Ins | **⚫** | **⚫** | **⚫** | **⚫** |
| 91 | *Hirundo rustica* | Hirundinidae | 20 | LC | intercontinental migrant | grass | Ins | **⚫** | **⚫** | **x** | **x** |
| 92 | *Icterus abeillei* | Oriolidae | 33 | LC | intercontinental migrant | forest | Disp | **⚫** | **x** | **x** | **x** |
| 93 | *Ispidina picta* | Alcedinidae | 16 | LC | intra african migrant | forest | Ins | **⚫** | **x** | **x** | **⚫** |
| 94 | *Jynx ruficollis* | Picidae | 59 | LC | resident | grass | Ins | **x** | **x** | **⚫** | **x** |
| 95 | *Kaupifalco monogrammicus* | Accipitridae | 246 | LC | resident and some local movement | forest | Pred | **x** | **x** | **⚫** | **x** |
| 96 | *Lagonosticta rubricata* | Estrildidae | 13 | LC | resident | forest | Disp | **⚫** | **x** | **x** | **x** |
| 97 | *Lamprotornis nitens* | Sturnidae | 105 | LC | resident | savanna | Disp | **⚫** | **⚫** | **⚫** | **⚫** |
| 98 | *Laniarius ferrugineus* | Malaconotidae | 69 | LC | resident | forest | Ins | **⚫** | **⚫** | **⚫** | **⚫** |
| 99 | *Lanioturdus torquatus* | Malaconotidae | 28 | LC | resident and some local movement | forest | Disp | **⚫** | **⚫** | **⚫** | **⚫** |
| 100 | *Lanius collaris* | Laniidae | 52 | LC | resident and some local movement | forest | Ins | **⚫** | **⚫** | **⚫** | **⚫** |
| 101 | *Lanius collurio* | Laniidae | 28 | LC | resident, intra african migrant and intercontinental migrant | savanna | Ins | **x** | **⚫** | **x** | **x** |
| 102 | *Lissotis melanogaster* | Otididae | 2700 | LC | resident and some local movement | savanna | Ins | **x** | **x** | **⚫** | **x** |
| 103 | *Lophaetus occipitalis* | Accipitridae | 1065 | LC | resident and some local movement | forest | Pred | **⚫** | **⚫** | **⚫** | **⚫** |
| 104 | *Lophoceros alboterminatus* | Bucerotidae | 332 | LC | intra african migrant | forest | Ins | **⚫** | **⚫** | **⚫** | **⚫** |
| 105 | *Lophoceros nasutus* | Bucerotidae | 233 | LC | resident and intra african migrant | savanna | Ins | **x** | **x** | **⚫** | **x** |
| 106 | *Lybius torquatus* | Lybiidae | 54 | LC | resident | forest | Disp | **⚫** | **⚫** | **⚫** | **⚫** |
| 107 | *Macronyx capensis* | Motacillidae | 44 | LC | resident | grass | Ins | **x** | **x** | **⚫** | **x** |
| 108 | *Macronyx croceus* | Motacillidae | 46 | LC | resident | grass | Ins |  | **⚫** | **⚫** | **x** |
| 109 | *Megaceryle maxima* | Cerylidae | 426 | LC | resident and intra african migrant | wetland | Pred | **⚫** | **⚫** | **x** | **⚫** |
| 110 | *Melaenornis pammelaina* | Muscicapidae | 30 | LC | resident | forest | Ins | **⚫** | **⚫** | **⚫** | **⚫** |
| 111 | *Melaenornis silens* | Muscicapidae | 26 | LC | resident and some local movement | savanna | Ins | **⚫** | **⚫** | **⚫** | **⚫** |
| 112 | *Melaniparus niger* | Paridae | 22 | LC | resident and some local movement | savanna | Ins | **⚫** | **x** | **⚫** | **⚫** |
| 113 | *Melierax canorus* | Accipitridae | 1300 | LC | resident and some local movement | savanna | Pred | **⚫** | **x** | **x** | **x** |
| 114 | *Merops pusillus* | Meropidae | 15 | LC | resident and some local movement | savanna | Ins | **x** | **x** | **⚫** | **x** |
| 115 | *Milvus aegyptius* | Accipitridae | 940 | NE | resident, some local movement and altitudinal migrant | forest | Ins | **x** | **⚫** | **x** | **x** |
| 116 | *Milvus migrans* | Accipitridae | 730 | LC | resident and intercontinental migrant | generalist | Scav | **⚫** | **x** | **⚫** | **⚫** |
| 117 | *Mirafra africana* | Alaudidae | 55 | LC | resident | grass | Ins | **x** | **x** | **⚫** | **x** |
| 118 | *Monticola rupestris* | Muscicapidae | 56 | LC | resident and altitudinal migrant | desert | Ins | **⚫** | **x** | **⚫** | **x** |
| 119 | *Motacilla aguimp* | Motacillidae | 27 | LC | resident | wetland | Ins | **x** | **⚫** | **⚫** | **⚫** |
| 120 | *Motacilla capensis* | Motacillidae | 21 | LC | resident and some local movement | wetland | Ins | **⚫** | **⚫** | **⚫** | **⚫** |
| 121 | *Motacilla flava* | Motacillidae | 18 | LC | Intercontinental migrant | grass | Ins | **x** | **x** | **⚫** | **x** |
| 122 | *Nectarinia famosa* | Nectariniidae | 14 | LC | nomad, some local movement and altitudinal migrant | fynbos | Poll | **⚫** | **⚫** | **x** | **⚫** |
| 123 | *Neotis denhami* | Otididae | 10000 | NT | resident and intra african migrant | grass | Ins | **x** | **x** | **⚫** | **x** |
| 124 | *Oena capensis* | Columbidae | 41 | LC | resident and nomad | savanna | Disp | **x** | **⚫** | **x** | **⚫** |
| 125 | *Oenanthe pileata* | Muscicapidae | 28 | LC | resident and intra african migrant | grass | Ins | **x** | **x** | **x** | **⚫** |
| 126 | *Onychognathus morio* | Sturnidae | 155 | LC | resident and nomad | generalist | Disp | **⚫** | **⚫** | **⚫** | **⚫** |
| 127 | *Oriolus larvatus* | Oriolidae | 71 | LC | resident and some local movement | forest | Disp | **⚫** | **⚫** | **⚫** | **⚫** |
| 128 | *Parus niger* | Paridae | 22 | LC | resident | forest | Ins | **x** | **x** | **⚫** | **x** |
| 129 | *Passer domesticus* | Passeridae | 40 | LC | resident | generalist | Disp | **⚫** | **x** | **x** | **x** |
| 130 | *Phoeniculus purpureus* | Phoeniculidae | 99 | LC | resident | savanna | Ins | **⚫** | **⚫** | **⚫** | **⚫** |
| 131 | *Phyllastrephus terrestris* | Pycnonotidae | 47 | LC | resident | forest | Ins | **x** | **⚫** | **x** | **⚫** |
| 132 | *Plectropterus gambensis* | Anatidae | 4480 | LC | nomad | wetland | Disp | **x** | **x** | **⚫** | **x** |
| 133 | *Ploceus bicolor* | Ploceidae | 47 | LC | resident | forest | Ins | **⚫** | **⚫** | **x** | **⚫** |
| 134 | *Ploceus capensis* | Ploceidae | 52 | LC | resident and some local movement | grass | Ins | **⚫** | **⚫** | **x** | **⚫** |
| 135 | *Ploceus cucullatus* | Ploceidae | 45 | LC | resident and some local movement | savanna | Disp | **x** | **x** | **x** | **⚫** |
| 136 | *Ploceus subaureus* | Ploceidae | 45 | LC | resident and some local movement | wetland | Ins | **⚫** | **⚫** | **x** | **⚫** |
| 137 | *Ploceus velatus* | Ploceidae | 34 | LC | resident and some local movement | savanna | Disp | **⚫** | **⚫** | **x** | **x** |
| 138 | *Pogoniulus pusillus* | Lybiidae | 17 | LC | resident | forest | Disp | **x** | **x** | **⚫** | **x** |
| 139 | *Pogonocichla stellata* | Muscicapidae | 21 | LC | resident and altitudinal migrant | forest | Disp | **⚫** | **x** | **x** | **x** |
| 140 | *Polyboroides typus* | Accipitridae | 774 | LC | resident and nomad | forest | Pred | **x** | **x** | **⚫** | **x** |
| 141 | *Prinia subflava* | Cisticolidae | 9 | LC | resident and altitudinal migrant | forest | Ins | **⚫** | **x** | **⚫** | **x** |
| 142 | *Prodotiscus regulus* | Indicatoridae | 14 | LC | resident and some local movement | forest | Ins | **⚫** | **x** | **x** | **x** |
| 143 | *Promerops gurneyi* | Promeropidae | 47 | LC | resident and altitudinal migrant | fynbos | Poll | **x** | **x** | **⚫** | **x** |
| 144 | *Psalidoprocne pristoptera* | Hirundinidae | 13 | LC | resident and altitudinal migrant | forest | Ins | **x** | **x** | **⚫** | **⚫** |
| 145 | *Pternistis adspersus* | Phasianidae | 635 | LC | resident | forest | Ins | **x** | **⚫** | **x** | **x** |
| 146 | *Pternistis afer* | Phasianidae | 765 | LC | resident | forest | Disp | **x** | **⚫** | **x** | **x** |
| 147 | *Pternistis swainsonii* | Phasianidae | 875 | LC | resident | forest | Disp | **⚫** | **⚫** | **⚫** | **⚫** |
| 148 | *Ptyonoprogne fuligula* | Hirundinidae | 16 | LC | intercontinental migrant | generalist | Ins | **⚫** | **x** | **x** | **x** |
| 149 | *Pycnonotus tricolor* | Pycnonotidae | 31 | LC | resident | forest | Disp | **⚫** | **⚫** | **⚫** | **⚫** |
| 150 | *Sagittarius serpentarius* | Sagittariidae | 3410 | LC | resident and nomad | grass | Scav | **x** | **x** | **⚫** | **x** |
| 151 | *Saxicola torquatus* | Muscicapidae | 17 | LC | resident and altitudinal migrant | grass | Ins | **⚫** | **x** | **⚫** | **x** |
| 152 | *Scleroptila levaillantii* | Phasianidae | 515 | LC | resident | forest | Ins | **x** | **x** | **⚫** | **x** |
| 153 | *Scopus umbretta* | Scopidae | 430 | LC | resident and nomad | wetland | Ins | **⚫** | **⚫** | **x** | **x** |
| 154 | *Spermestes cucullatus* | Estrildidae | 12 | LC | resident | forest | Disp | **⚫** | **x** | **x** | **x** |
| 155 | *Spermestes fringilloides* | Estrildidae | 14 | LC | resident and nomad | forest | Disp | **x** | **x** | **⚫** | **x** |
| 156 | *Spilopelia senegalensis* | Columbidae | 102 | LC | resident and some local movement | forest | Disp | **⚫** | **⚫** | **x** | **x** |
| 157 | *Stephanoaetus coronatus* | Accipitridae | 5000 | NT | resident and some local movement | forest | Pred | **x** | **x** | **x** | **⚫** |
| 158 | *Streptopelia capicola* | Columbidae | 153 | LC | resident and some local movement | forest | Disp | **⚫** | **⚫** | **⚫** | **⚫** |
| 159 | *Streptopelia decipiens* | Columbidae | 230 | LC | resident and some local movement | savanna | Disp | **⚫** | **⚫** | **x** | **⚫** |
| 160 | *Streptopelia semitorquata* | Columbidae | 252 | LC | resident and some local movement | forest | Disp | **x** | **⚫** | **⚫** | **x** |
| 161 | *Sturnus vulgaris* | Sturnidae | 100 | LC | resident and some local movement | generalist | Disp | **⚫** | **⚫** | **x** | **⚫** |
| 162 | *Sylvia borin* | Sylviidae | 21 | LC | intra african migrant | forest | Ins | **⚫** | **x** | **x** | **x** |
| 163 | *Tachymarptis melba* | Apodidae | 77 | LC | resident and intra african migrant | generalist | Ins | **⚫** | **x** | **⚫** | **⚫** |
| 164 | *Tadorna cana* | Anatidae | 1171 | LC | resident and some local movement | wetland | Scav | **x** | **⚫** | **x** | **x** |
| 165 | *Tauraco corythaix* | Musophagidae | 306 | LC | resident and some local movement | forest | Disp | **⚫** | **⚫** | **⚫** | **⚫** |
| 166 | *Tchagra senegalus* | Malaconotidae | 52 | LC | resident | savanna | Ins | **x** | **⚫** | **x** | **x** |
| 167 | *Telophorus zeylonus* | Malaconotidae | 76 | LC | resident and altitudinal migrant | grass | Ins | **⚫** | **⚫** | **⚫** | **x** |
| 168 | *Terpsiphone viridis* | Monarchidae | 14 | LC | intra african migrant | forest | Ins | **⚫** | **⚫** | **⚫** | **⚫** |
| 169 | *Thamnolaea cinnamomeiventris* | Muscicapidae | 51 | LC | resident and altitudinal migrant | savanna | Ins | **x** | **x** | **⚫** | **x** |
| 170 | *Trachyphonus vaillantii* | Lybiidae | 74 | LC | resident and some local movement | savanna | Disp | **x** | **x** | **⚫** | **x** |
| 171 | *Treron calvus* | Columbidae | 285 | LC | resident and some local movement | forest | Disp | **⚫** | **⚫** | **⚫** | **⚫** |
| 172 | *Turdus merula* | Turdidae | 135 | LC | intercontinental migrant | forest | Disp | **x** | **⚫** | **x** | **x** |
| 173 | *Turdus olivaceus* | Muscicapidae | 81 | LC | resident and altitudinal migrant | forest | Disp | **⚫** | **⚫** | **⚫** | **⚫** |
| 174 | *Turtur tympanistria* | Columbidae | 85 | LC | resident and some local movement | forest | Disp | **x** | **x** | **x** | **⚫** |
| 175 | *Upupa africana* | Upupidae | 67 | LC | resident, nomad and intra african migrant | forest | Ins | **x** | **x** | **x** | **⚫** |
| 176 | *Urocolius indicus* | Collidae | 56 | LC | resident and some local movement | forest | Disp | **x** | **⚫** | **x** | **x** |
| 177 | *Vidua macroura* | Viduidae | 19 | LC | resident and nomad | forest | Disp | **x** | **x** | **⚫** | **x** |
| 178 | *Zosterops virens* | Zosteropidae | 11 | LC | resident and nomad | forest | Disp | **⚫** | **⚫** | **x** | **⚫** |

Table S2. The Multiple Correspondence Analysis (MCA) showing the eigen values, percentage of variance and cumulative percentage of variance.

| **Dimensions** | **Eigenvalue** | **Percentage of variance** | **Cumulative % of variance** |
| --- | --- | --- | --- |
| Dim.1 | 0.51451 | 27.25173 | 27.25173 |
| Dim.2 | 0.41363 | 21.90835 | 49.16008 |
| Dim.3 | 0.28726 | 15.21518 | 64.37527 |
| Dim.4 | 0.25000 | 13.24152 | 77.61679 |
| Dim.5 | 0.21533 | 11.40491 | 89.02170 |
| Dim.6 | 0.12735 | 6.74516 | 95.76685 |
| Dim.7 | 0.07992 | 4.23315 | 100 |

Table S3: The coordinate table showing the position of each variable category in the dimension space. Bold values indicate variables with a significant and strong effect on the dimensions. Larger negative and positive values also represent a strong correlation.

|  | **Variables** | **Dim 1** | **Dim 2** | **Dim 3** | **Dim 4** | **Dim 5** |
| --- | --- | --- | --- | --- | --- | --- |
| Bird body size classes | Largebirds | **-0.71092** | 0.41273 | -0.02134 | 0.095212 | 0.031326 |
|  | Medium birds | 0.016987 | **-0.55266** | -0.18579 | **-0.54449** | 0.281796 |
|  | Small birds | 0.068587 | -0.66536 | 0.357889 | **0.619729** | -0.32265 |
|  | Tiny birds | **0.84155** | **0.496101** | -0.01805 | 0.033014 | -0.11842 |
| Bird ecological roles | Carrion | -1.5909 | **1.673706** | **2.932888** | -0.39809 | **1.14253** |
|  | Dispersers | -0.23753 | -0.32986 | -0.01358 | 0.437257 | 0.252323 |
|  | Insectivores | 0.232627 | -0.14252 | 0.087963 | -0.37499 | -0.24395 |
|  | Pollinators | **1.838871** | **1.343869** | -0.09863 | **0.824547** | 0.284976 |
|  | Predators | **-0.93613** | **0.969567** | **-0.76127** | -0.11974 | -0.04511 |
